# Usefulness of bilateral plate fixation for periprosthetic distal femur fracture after total knee arthroplasty

**DOI:** 10.1016/j.ijscr.2020.01.043

**Published:** 2020-02-06

**Authors:** Kwangkyoun Kim

**Affiliations:** Department of Orthopaedic Surgery, Konyang Unversity Hospital, Gasoowon-dong, Seo-gu, Daejeon, Republic of Korea

**Keywords:** Knee, Periprosthetic fracture, Total knee arthroplasty, Locking compression plate

## Abstract

**Introduction:**

Recently, good results of locking compression plate (LCP) have been reported in the case of fixation using plate, but when fractures extend too distal over the proximal border of femoral component, operations are highly challenging.

**Presentation of case:**

Author reports two cases that couldn't get reliable fixation by LCP alone because of poor bone quality with chronic infection and far juxta-fracture of prosthesis. Fractures were fixed to the medial and lateral sides of the distal femur with LCPs and were healed successfully.

**Discussion:**

Indirect healing (endochondral bone formation) using anatomical alignment and bridge plating using LCP is usually used rather than direct healing (intramembranous) through anatomical reduction and rigidly stable fixation for periprosthetic distal femoral fracture. Proper flexible fixation is helpful for indirect bone healing through the callus formation, but too flexible fixation cannot maintain fracture fragments until the callus formation provides sufficient stability. Medial parapatella approach for additional medial side plating have many advantages that it can confirm the screw length which not penetrate intercondylar box or medial cortex, and can check component stability, rotation, wear of the polyethylene insert.

**Conclusion:**

Bilateral plate fixation through additional medial parapatella approach is useful method for obtaining secure fixation to poor bone quality or extremely distal femoral periprosthetic fracture.

## Highlights

• Author got reliable fixation with double plating in situation, could not get secure stability by unilateral lateral plating.
• Authors used medial parapatella approach for placing an additional plate on the medial side of distal femur.
• Parapatella approach for additional plating can check screw length, femoral component stability and rotation, and polyethylene insert wear.

## 1 Introduction

As our society being aged, the rates of total knee arthroplasty (TKA) grow up, and this phenomenon result in increase of periprosthetic fractures after TKA [1,2]. Periprosthetic distal femur fracture after TKA usually can be operated by intramedullary nailing, plating or revision arthroplasty [[3], [4], [5]]. Over recent years, locking compression plate (LCP) has been shown good clinical results for the management of supracondylar femoral fracture. But if fractures extend too distal over the proximal border of femoral component operations are highly challenging. Reliable fixation can be difficult to achieve, owing to interference of the prosthesis and presence of poor bone stock. Author reports two cases that could not get reliable fixation by lateral locking plate alone because of poor bone quality and far juxta-fracture of prosthesis.

This work was reported in line with the SCARE criteria [6].

### 1.1 Case report 1

**Fig. 1:**
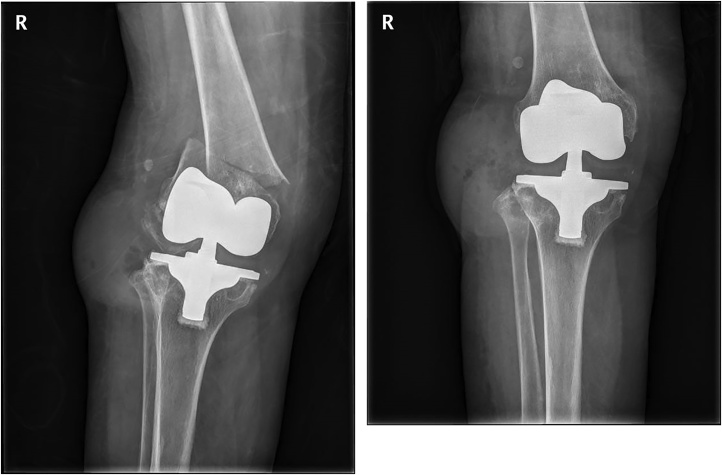
A) Radiograph showed a complete displaced fracture, which was not seen at visiting an outpatient clinic. (B) radiograph taken at outpatient clinic showed a huge soft tissue mass shadow on antero-lateral aspect of knee.

**Fig. 2:**
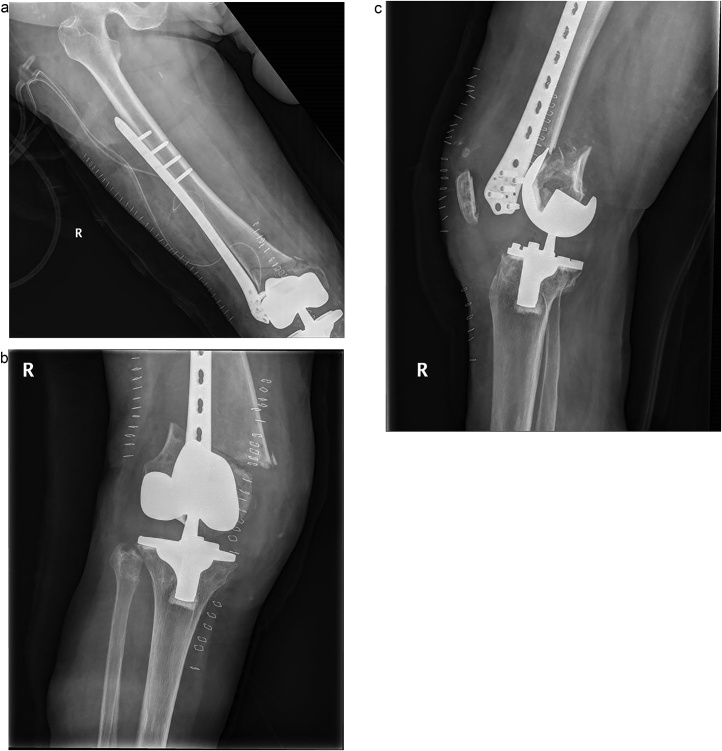
A) Postoperative radiography showed a reduction of displaced fracture and fixaion with LCP on the lateral side of femur (B–C) at postoperative 9th day, radiograph showed the reduction loss of fracture and fixation failure.

**Fig. 3:**
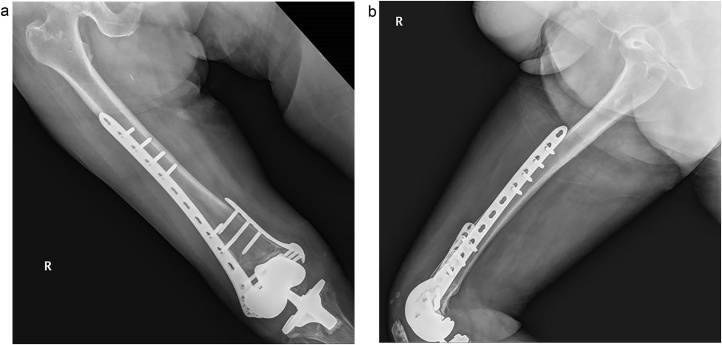
Radiographs at postoperative 6 months, showed a complete fracture healing with dual plating. (A) anterioposterior radiograph (B) lateral radiograph.

A 76 years old woman brought emergency center by painful swelling in right knee caused by falling down from wheelchair. Right distal femur showed a periprosthetic fracture of the TKR operated 15 years ago (Fig. 1A). Two days ago, she visited an outpatient department because of serous discharge from anterolateral aspect and varus instability of right knee. Radiographs, checked outpatient clinic, showed a huge mass like soft tissue lesion on anterolateral aspect of knee (Fig. 1B). Joint fluid analysis showed that white blood cell count was 1728 and CRP 0.5 mg/dl. Culture result showed no growth for 48 hours. We operated with lateral locking plate (Synthes, Switzerland) and changing a bearing insert with debridement of the huge mass and sinus track (Fig. 2A). At post-operative 7 days, candida was detected from the culture obtained at operation, equivalent to catheter tip culture. So after then, we use anti-fungal antibiotics. In 9th post-operative day, author felt the abnormal motion at fracture site at changing the dressing gauzes. We found fixation failure and reduction loss on the radiography (Fig. 2B, C). Author removed the failed lateral plate, and reduced fracture and placed the new lateral plate again. Because of poor bone quality (T-score; total hip = −4.2 g/cm^2^) and chronic fungal infection, we could not acquire rigid fixation after lateral locking plating. In addition, author cannot use enough length of screws for penetrating the far cortex, because femur prosthesis was a posterior cruciate ligament scarifying type with a closed box. So we placed a LCP on the medial side additionally. After 6 months later, fracture was healed and infection was controlled (Fig. 3).

### 1.2 Case report 2

**Fig. 4:**
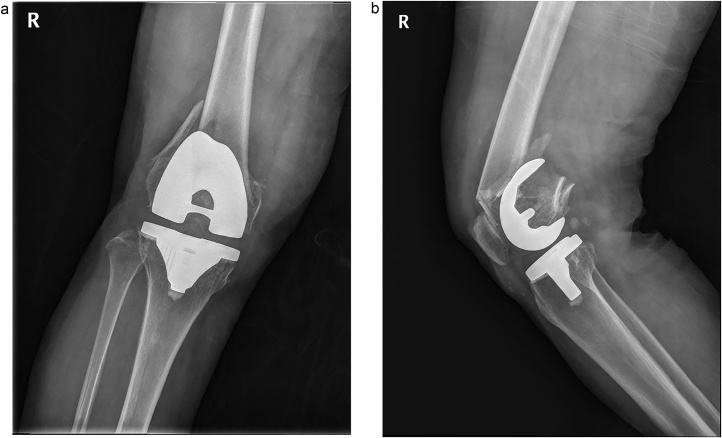
Preoperative radiographs showed that fracture line extends more distal than proximal border of an implant. (A) anterioposterior radiograph (B) lateral radiograph.

A 71-year-old woman brought emergency center by painful swelling on right knee after slipping down from a scooter. Periprosthetic fracture was shown in right distal femur. Fracture was severely comminuted on medial and lateral side and fracture line extended to distal to proximal femoral border of prosthesis (Fig. 4).

**Fig. 5:**
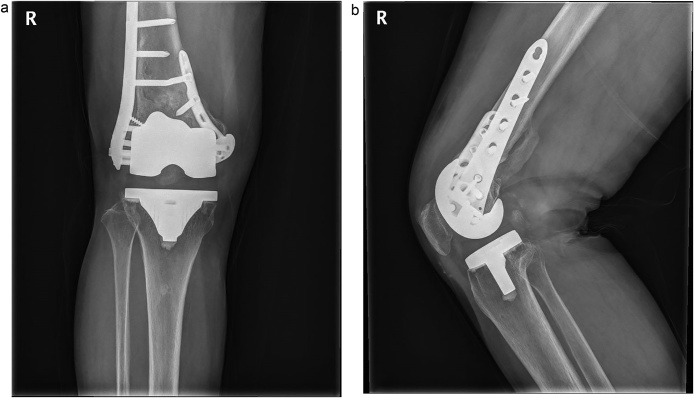
In the postoperative 6 months, radiographs show complete fracture healing after dual plating on both sides. (A) anterioposterior radiograph (B) lateral radiograph.

Author used a minimally invasive lateral approach. An about 4 cm incision was made over the lateral femoral condyle, directly over the fracture site and point of entry of the LCP. After reduction of fracture temporally as necessary, the plate was passed through the incision proximally, beneath the vastus lateralis. Using anatomical landmarks and C-arm imaging, placed the plate on the lateral condyles. Because fracture was very severely comminuted on both medial and lateral sides and too distal over the proximal border of femoral component, there was not enough space for placing screws with secure on the distal fragment of the fracture. It made the lateral locking plating alone to be not secure the fracture. So, additional medial side plate was need to get firm fixation. We made another midline skin incision along the previous incision scar for a total knee replacement, and made a medial parapatella approach. We retracted patella and confirmed fracture reduction and femoral component rotation and alignment. We checked the screws which not penetrate intercondylar box with native eye. After conforming secure fixation of fracture, we checked the stability of knee. The polyethylene insert was nearly wasted. So, we exchanged it with new one. Wound were closed layer by layer. Post-operative 6 months, radiography shows complete healing of the fracture (Fig. 5).

## 2 Discussion

Recently, many studies have shown results of the LCP fixation and retrograde intramedullary nail (RIMN) fixation. LCP and RIMN have been reported to yield better outcomes than traditional non-locking plates [3]. Data from large retrospective cohorts [3,[7], [8], [9]] (summarized in Table 1) suggest that there were no significant differences in clinical outcomes, including time to union and union rates, in patients who underwent LCP and RIMN fixation for periprosthetic fractures of the distal femur after TKA. However, RIMN demonstrated a significantly higher malunion rate when compared with locked plating.

**Table 1:** Summary of table of the most important published studies on the osteosynthesis of periprosthetic distal femur fractures after total knee arthroplasty.

Author/year	Enrollment	Main findings
Ebraheim at al 2015 [7]	488	The most frequent type of periprosthetic distal femur fracture after total knee arthroplasty was Rorabeck type II. The most common treatments for these types of fractures are locked plating and intramedullary nailing, with similar healing rates of 87% and 84%, respectively. However, the complication rate for locked plating (35 %) was lower than for intramedullary nailing (53 %).
Meneghini et al 2014 [3]	85	Seventy-one of 85 knees (83.5 %) went on to union at an average of 16 weeks. There were 2 (9 %) nonunions in the IM nail group and 12 nonunions or delayed unions (19 %) in the locked plate group (P = 0.34). There was no difference in time to union between groups (P = 0.64).
Matlovich et al 2017 [8]	57	There was no statistical difference between groups in the mean time to fully weight bearing, the incidence of postoperative pain, range of motion, use of gait aids, time to full radiographic union, or the overall radiographic alignment of a healed fracture (P > 0.05). Comparison based on fracture location yielded similar outcomes. Nonunion was only demonstrated in the IM nail cohort, particularly for fractures below the TKA flange (n = 2).
Ristevski et al 2014 [9]	719	Comparison of locked plating and retrograde intramedullary nailing (RIMN) showed no significant differences with regard to nonunion rates (OR = 0.39, P = 0.09). However, RIMN demonstrated a significantly higher malunion rate when compared with locked plating (OR = 2.37, P = 0.02).

The characteristic stable plate-screw connection of LCP reduces the risk of secondary loss of reduction, preservation of the blood supply to soft tissues and bone, providing absolute or relative stability, and improvement of the fixation in osteoporotic bone. Nevertheless, distal femoral periprosthetic fracture makes osteosynthesis difficult to obtain. Most patients with periprosthetic fractures developed at older, with osteoporosis following. It can be also difficult to achieve reliable fixation, owing to interference of the prosthesis, poor bone stock due to loss during previous surgery, and very close proximity of fracture to the prosthesis [10,11].

In terms of bone healing mechanism, the distal femur anatomy, sometime fracture lines are covered by prosthesis, so it is difficult to obtain accurate anatomical reduction due to lack of anatomical landmarks, as well as previous TKA may impair blood supply around the bone [[12], [13], [14]]. Therefore, indirect healing (endochondral bone formation) using anatomical alignment and bridge plating using LCP is usually used rather than direct healing (intramembranous) through anatomical reduction and rigidly stable fixation. There is limit to simple radiography to verify that the prosthesis was correctly aligned with the previous TKA, so it is necessary to check the alignment status of the prosthesis with three dimensional computed tomography before surgery.

In our first case, we couldn’t get enough stability by only lateral locking plating. Ricci et al. [5] reported that implant failure of locked plate fixation of distal femur was related to open fracture, smoking, increased body mass index, and shorter plate length. In this case, causes of the failure were the interference of closed box of the femur prosthesis and poor bone quality, which were low bone mineral density (T-score; total hip = −4.2 g/cm^2^) and chronic fungal infection. It is necessary to know that only lateral plating cannot be enough to reliable fixation, so bilateral locking plating was considered for fracture too distal over the prosthesis and poor bone quality. In terms of bone healing mechanism, surgery was performed with indirect healing concept (callus formation) using anatomical alignment and bridge plating with LCP, but failed due to too flexible and insufficient fixation. This case showed that proper flexible fixation is helpful for indirect bone healing through the callus formation, but too flexible fixation cannot maintain fracture fragments until the callus formation provides sufficient stability.

In our second case, fracture line was too distal over the prosthesis. Reflecting difficulty about too distal fracture, Su et al. [15] established three periprosthetic fracture types according to the most distal extent of the fracture relative to proximal border of the femoral component. Type I fractures are located proximal to the component, Type II extend from the proximal aspect anterior prosthetic flange proximally, and Type III extend distally beyond the proximal border of the femoral component.

Authors used medial parapatella approach for placing an additional plate on the medial side of distal femur. In use of medial approach, careful observation is needs not to injury neurovascular structures, because it's already rebutted state. Medial parapatella approach have many advantages that it can confirm the screw length which not penetrate intercondylar box or medial cortex, and can check component stability, rotation, wear of the polyethylene insert. It is easy for polyethylene insert exchange when it was wasted, and for thorough debridement suspected infection.

Considering the disadvantages of the medial parapatella approach used in TKA, additional medial parapatella approach for medial side plating may be to reduce quadriceps muscle strength after surgery, especially if the medial parapatella approach was previously used in TKA. An incision in the vicinity of the patella can also cause the blood supply impairment to the patella, avascular necrosis, patella maltracking, or anterior knee pain [16,17]. In our two cases, at the final follow up, quadriceps muscle strength was recovered to grade V and there were no complications associated patella. However, more cases need to be collected and more research is needed on these potential complications.

## 3 Conclusion

Bilateral plate fixation through additional medial parapatella approach is useful method for obtaining secure fixation to poor bone quality or extremely distal femoral periprosthetic fracture.

“Written informed consent was obtained from the patient for publication of this case report and accompanying images. A copy of the written consent is available for review by the Editor-in-Chief of this journal on request”.

## Sources of funding

I have nothing to declare.

## Ethical approval

The patient provided consent for data concerning this case to be submitted for publication and approved by the internal review board of our institution (KYUH 2019-02-001).

## Consent

Written informed consent was obtained from the patient for publication of this case report and accompanying images. I attached a informed consent.

## Author contribution

Kwangkyoun Kim did all of study concept or design, data collection, data analysis or interpretation, writing the paper.

## Registration of research studies

This case report is not a research involving human participants.

## Guarantor

Kwangkyoun is the guarantor responsibility for the work and/or the conduct of the study, had access to the data, and controlled the decision to publish.

## Provenance and peer review

Not commissioned, externally peer-reviewed.

## Declaration of Competing Interest

I have nothing to declare.
